# Free Vibration Analysis of a Tunable Micro-Fabrication Device Comprising Asymmetric L-Shaped Membranes

**DOI:** 10.3390/polym15102293

**Published:** 2023-05-12

**Authors:** Cheng-Hua Xiong, Lian-Gui He, Kao-Hao Chang, Chang-Wei Huang

**Affiliations:** 1 College of Civil Engineering and Architecture, Sanming University, Jing Dong Road, Sanming 365004, China; 20190961213@fjsmu.edu.cn (C.-H.X.); hlg@fjsmu.edu.cn (L.-G.H.); 2 Key Laboratory of Engineering Material & Structure Reinforcement in Fujian Province College, Sanming University, Sanming 365004, China; 3 Department of Civil Engineering, National Kaohsiung University of Science and Technology, Kaohsiung City 807618, Taiwan; 4 Department of Civil Engineering, Chung Yuan Christian University, Taoyuan City 320314, Taiwan

**Keywords:** free vibration, asymmetric L-shaped membrane, semi-analytical treatment, frequency-tunable membrane sensors

## Abstract

Membrane sensors have been widely used in various fields owing to their multifunctionality and cost-effectiveness. However, few studies have investigated frequency-tunable membrane sensors, which could enable versatility in the face of different device requirements while retaining high sensitivity, fast response times, and high accuracy. In this study, we propose a device comprising an asymmetric L-shaped membrane with tunable operating frequencies for microfabrication and mass sensing applications. The resonant frequency could be controlled by adjusting the membrane geometry. To fully understand the vibration characteristics of the asymmetric L-shaped membrane, the free vibrations of the membrane are first solved by a semi-analytical treatment combining domain decomposition and variable separation methods. The finite-element solutions confirmed the validity of the derived semi-analytical solutions. Parametric analysis results revealed that the fundamental natural frequency decreases monotonically with the increase in length or width of the membrane segment. Numerical examples revealed that the proposed model can be employed to identify suitable materials for membrane sensors with specific frequency requirements under a given set of L-shaped membrane geometries. The model can also achieve frequency matching by changing the length or width of membrane segments given a specified membrane material. Finally, performance sensitivity analyses for mass sensing were carried out, and the results showed that the performance sensitivity was up to 0.7 kHz/pg for polymer materials under certain conditions.

## 1. Introduction

In the past few decades, polymer membranes have been widely used to manufacture vibration sensors owing to their mechanical flexibility, high sensitivity, cost-effectiveness, and facile integration into electronic circuits. These sensors, comprising a flexible membrane, detect changes in the surrounding environment based on changes in the vibration characteristics. Owing to the high sensitivity of membrane vibrations to changes in the surrounding environment, these sensors can detect small changes in temperature, pressure, and other physical parameters. As a result, polymer-membrane vibration sensors are applied in numerous fields, including structural health monitoring, the automotive industry, and medical diagnostics [1,2,3,4,5].

Understanding the vibration characteristics of membranes is required to extend the applications of membrane sensors. Several studies have documented the natural frequencies of homogeneous membranes [6,7]. Generally, exact analytical solutions are limited to simple geometries such as rectangles, circles, sectors, ellipses, and isosceles right triangles [8,9,10]. Various approximate approaches have been developed to solve problems involving polygonal or compound geometries with Dirichlet, Neumann, or mixed boundary conditions [11,12,13,14,15]. As alternative practical routes, numerical schemes such as the finite-difference, finite-element (FE), boundary-element (BE), and mesh-free methods have been reported [16,17,18,19,20,21,22,23,24]. Recently, novel numerical methods, such as the Galerkin [25] and Bezier [26] methods, have been shown to attain higher stability and accuracy than other numerical methods.

Among previous research topics, homogeneous L-shaped membranes are apparently favored, especially symmetric ones [27,28]. Associated eigenvalues are often used as validation criteria or cross-reference sources [29,30,31,32,33,34,35,36,37,38,39]. Unfortunately, the theoretical solution for the corresponding free vibration problems is seemingly difficult. The most commonly used method (i.e., the variable separation method) is only feasible for simple geometric membranes whose boundaries fit perfectly into a particular separable coordinate system, such as circular and elliptical membranes [8]. Although the geometries of L-shaped membranes inherently consist of rectangles, exact analytical solutions for symmetric and asymmetric cases are not readily available. This is because the contours of L-shaped geometries only partially conform to a Cartesian rectangular coordinate system.

As frequency-tunable membrane sensors demonstrate more comprehensive applications (e.g., biomedical sensing, environmental monitoring, and industrial process control), a conceptual model for a tunable microfabricated device comprising asymmetric L-shaped membranes is proposed herein. First, a Fourier series solution is derived, which can be used as an alternative to an exact analytical solution. The region-matching technique combines the domain decomposition method and the variable separation method. When the appropriate auxiliary boundary is selected, the eigenfunctions in each subregion inherently satisfy most parts of the clamped boundary conditions. This semi-analytical nature permits relatively fast convergence and relatively high accuracy of the present results compared to those obtained using commercial FE software Abaqus [40].

This serves as the cornerstone of our investigation into frequency-tunable sensor design. The operating frequency of these membrane-based sensors can be tuned by either material selection or membrane geometry. The semi-analytical solution presented above is used to determine a suitable material for a membrane vibration sensor with a specific frequency requirement. In addition, frequency tuning is demonstrated by varying the length or width of the membrane segments.

## 2. Materials and Methods

**Figure 1:**
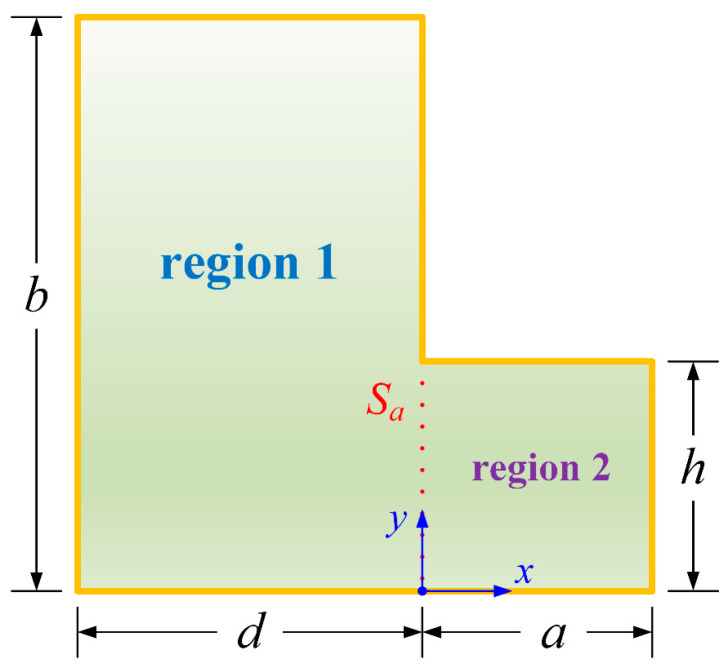
Geometric layout of the asymmetric membrane.

Consider a tunable microfabricated device comprising a stretched asymmetric L-shaped membrane clamped on all edges. Figure 1 shows the problem geometry. The thin elastic membrane is assumed to be homogeneous and is characterized by four parameters, *b*, *d*, *a*, and *h*, where *b* and *d* represent the length and width of the left-hand segment, and *a* and *h* represent those of the right-hand segment, respectively. The uniform tensile force per unit length is *T*, and the constant mass per unit area is *ρ*. The origin of the Cartesian coordinate system (*x*, *y*) is the vertical projection point of the concave corner on the underside of the membrane.

Based on the domain decomposition method, a vertical auxiliary boundary *S_a_* is introduced to divide the entire computational domain into two enclosed regions: regions 1 and 2 (see Figure 1). The small transverse motions of the membrane, denoted by *u_j_*, must obey the governing Helmholtz equations:

(1) $$\nabla^{2}u_{j}+k^{2}u_{j}=0,\;j=1,\;2,$$

where $\nabla^{2}$ is the two-dimensional Laplacian operator in the *x–y* plane, $k=\omega \sqrt{\rho /T}$ is the wavenumber, and *ω* is the angular natural frequency of vibration. The subscript *j*, where *j* = 1 and 2, represents the region number. The time-harmonic factor is understood throughout this section.

The zero-displacement boundary conditions are imposed along the edge of region 1,

(2) $$u_{1}(x,y)=0,\;y=0,\;-d\leq x\leq 0,$$

(3) $$u_{1}(x,y)=0,\;x=-d,\;0\leq y\leq b,$$

(4) $$u_{1}(x,y)=0,\;y=b,\;-d\leq x\leq 0,$$

(5) $$u_{1}(x,y)=0,\;x=0,\;h\leq y\leq b,$$

and along the edge of region 2,

(6) $$u_{2}(x,y)=0,\;y=0,\;0\leq x\leq a,$$

(7) $$u_{2}(x,y)=0,\;x=a,\;0\leq y\leq h,$$

(8) $$u_{2}(x,y)=0,\;y=h,\;0\leq x\leq a,$$

By applying the method of eigenfunction expansion, the displacement fields in regions 1 and 2, respectively, can be expressed as follows:

(9) $$u_{1}(x,\;y)=\sum_{n=1}^{\infty }A_{n}\frac{\sinh\left[\alpha_{n}(x+d)\right]}{\sinh(\alpha_{n}d)}\sin\left(\frac{n\pi }{b}y\right),$$

(10) $$u_{2}(x,\;y)=\sum_{n=1}^{\infty }B_{n}\frac{\sinh\left[\beta_{n}(x-a)\right]}{\sinh(\beta_{n}a)}\sin\left(\frac{n\pi }{h}y\right),$$

with

(11) $$\alpha_{n}=\sqrt{{\left(\frac{n\pi }{b}\right)}^{2}-k^{2}},$$

(12) $$\beta_{n}=\sqrt{{\left(\frac{n\pi }{h}\right)}^{2}-k^{2}},$$

where the expansion coefficients *A_n_* and *B_n_* are unknown. Notably, Equations (9) and (10) inherently satisfy the governing Equation (1) and most of the boundary conditions around the membrane edge, except those on *S_a_*.

Enforcing the displacement continuity condition on *S_a_* yields

(13) $$u_{1}(x,\;y)=u_{2}(x,\;y),\;x=0,\;0\leq y\leq h,$$

By multiplying Equation (13) by a sequence of sine functions and integrating over the appropriate intervals,

(14) $$\int_{0}^{b}u_{1}(0,\;y)\sin\left(\frac{q\pi }{b}y\right)dy=\int_{0}^{h}u_{2}(0,\;y)\sin\left(\frac{q\pi }{b}y\right)dy,\;q=1,\;2,\cdots ,$$

According to Equation (5), the result of integration from *h* to *b* vanishes. This implies that the upper limit of integration on the left-hand side of Equation (14) can be extended from *h* to *b*. Therefore, the orthogonal property of sine functions can be applied to Equation (14) directly. Consequently, the following relation holds:

(15) $$A_{n}=\frac{2}{b}\sum_{m=1}^{\infty }B_{m}I_{m,n}^{S},$$

where

(16) $$I_{m,n}^{S}=\left\{\;\begin{array}{cc} h/2, & mb=nh\\ -\frac{mh{\left(-b\right)}^{2}}{\pi \left[{\left(mb\right)}^{2}-{\left(nh\right)}^{2}\right]}\sin\left(\frac{n\pi }{b}h\right), & m\neq n \end{array},\right.$$

Similarly, by considering the slope continuity condition across *S_a_*,

(17) $$\frac{\partial u_{1}(x,\;y)}{\partial x}=\frac{\partial u_{2}(x,\;y)}{\partial x},\;x=0,\;0\leq y\leq h,$$

and applying successive sine functions, and integrating over the range [0, *h*],

(18) $$\int_{0}^{h}\frac{\partial u_{1}(0,\;y)}{\partial x}\sin\left(\frac{q\pi }{h}y\right)dy=\int_{0}^{h}\frac{\partial u_{2}(0,\;y)}{\partial x}\sin\left(\frac{q\pi }{h}y\right)dy,\;q=1,\;2,\cdots ,$$

By exploiting Equation (15) to eliminate the unknown coefficients *A_n_* and rearranging the results in a system of linear algebraic equations (with unknown coefficients *B_n_*), the following matrix form can be obtained:

(19) $$\left[{ℳ}_{i,j}(k)\right]\left\{B_{j}\right\}=\left\{0\right\},\;i=1,\;2,\cdots ,\;j=1,\;2,\cdots ,$$

with

(20) $${ℳ}_{i,j}(k)=\sum_{n=1}^{\infty }\alpha_{n}\coth(\alpha_{n}d)I_{i,n}^{S}I_{j,n}^{S}+\delta_{i,j}\beta_{i}\frac{bh\coth(a\beta_{i})}{4},$$

where *δ_i,j_* is the Kronecker delta function.

Clearly, Equation (19) constitutes a generalized matrix eigenvalue problem, as expected. Standard techniques can thus be used to evaluate the natural frequencies of the present membrane, which are related to the roots of the determinant equation given by

(21) $$\det\left[{ℳ}_{i,j}(k)\right]=0,$$

Once the eigenvalues *k* are found, the expansion coefficients *B_n_* can be evaluated using partitioned matrices and block multiplication (cf. Equation (19)). Therefore, the expansion coefficients *A_n_* are determined directly from Equation (15). Eventually, the natural modes (eigenmodes) can be obtained by Equations (9) and (10).

In Equation (20), the summation indices *n* and the weighting indices *i* and *j* are truncated after *N* terms. Hence, Equation (19) constitutes a system of *N* equations with *N* + 1 unknowns. The number of truncation terms in consideration depends only on the accuracy requirement.

## 3. Results

For nondimensionalization, the width of the right-hand segment *h* is taken as the characteristic length. The eigenvalues *k_i_* are given, in dimensionless form, as follows:

(22) $$k_{i}=h\omega \sqrt{\rho /T},\;i=1,\;2,\cdots ,$$

Before undertaking semi-analytical and FE modeling on L-shaped membranes, pertinent parameter settings adopted for symmetric and asymmetric cases are shown in Table 1 and Table 2.

### 3.1. Convergence Test for Semi-Analytical Modelling

At the initial stage of semi-analytical modelling, some numerical experiments are carried out to determine the convergence criterion of the series solution proposed. Table 3 displays the nine selected eigenvalues versus truncation indices *N* for the asymmetric case 1. As seen in Table 3, all the eigenvalues converged to at least 2-decimal-place accuracy at *N* = 20. Taking *N* = 500 guarantees 4-decimal-place accuracy. When *N* = 2000, all the computed results are the same as those at *N* = 500, implying that the present solution procedure remains numerically stable. This exemplifies the good performance of the proposed scheme.

### 3.2. Mesh Sensitivity Analysis for FE Modeling

**Figure 2:**
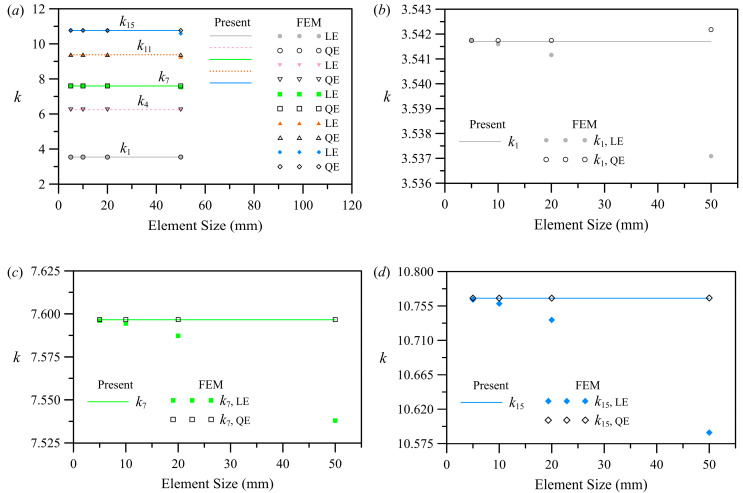
Selected eigenvalues versus the element size for the asymmetric case 1: (**a**) overall view for *k*_1_, *k*_4_, *k*_7_, *k*_11_, and *k*_15_; (**b**) close-up view for *k*_1_; (**c**) close-up view for *k*_7_; (**d**) close-up view for *k*_15_.

For the asymmetric case 1, the FE solution was used to cross-reference and validate the semi-analytical approach. To ensure the convergence of the FE simulation, mesh sensitivity analyses were performed by reducing the element size. Figure 2 shows the first, fourth, seventh, eleventh, and fifteenth eigenvalues obtained from the semi-analytical and FE solutions. Figure 2a shows an overall view of the five selected eigenvalues, while Figure 2b–d shows the magnified views of the first, seventh, and fifteenth eigenvalues. Notably, the solution domain is discretized in the FE analysis using three-dimensional linear and quadratic membrane elements (M3D4 and M3D8 in Abaqus [40]). The FE simulations were conducted with element numbers ranging from 874 to 120,000, and nodes ranging from 2763 to 361,601. The solid, dashed, and dotted lines represent the results of the derived series solution, while the solid and hollow symbols represent the FE solutions.

Figure 2a shows the results obtained from converged FE simulations. The results are in agreement with those obtained from the series solution when the characteristic element size is less than or equal to 20 mm. Clearly, from Figure 2b–d, the convergence rate of linear element (LE) results is slower than that of quadratic element (QE) results. The quadratic element was hence adopted for comparison with the semi-analytical solutions of 4-decimal-place accuracy, and a 5 mm element size was selected for later validation and parametric analyses.

### 3.3. Verification

To verify the proposed semi-analytical solution, the first 10 eigenvalues for the symmetric case were calculated and are shown in Table 4, as well as the results obtained from the FE solver, Abaqus [40], and those reported previously [17,27,39]. The consistency between the present results and those obtained from different numerical schemes is clear. Similarly, Table 5 shows the first fifteen eigenvalues of asymmetric case 1 (as defined in Table 1). The overall agreement remains quite good, which confirms the accuracy and reliability of the series solution derived here.

### 3.4. Effect of Geometric Parameters on Eigenvalues

**Figure 3:**
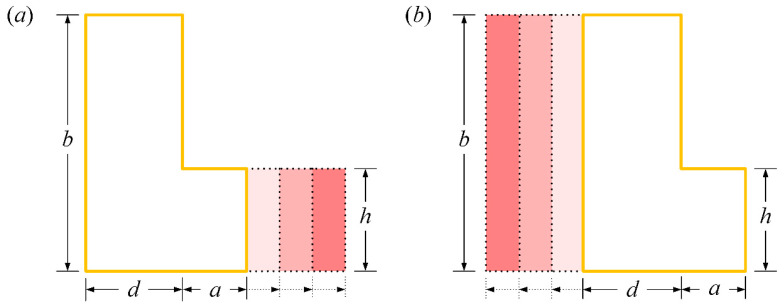
Actively reconfigurable L-shaped membranes: (**a**) tunable segment length; (**b**) tunable segment width.

A membrane-type vibration sensor able to actively shift the resonant frequency within a specific range is desired. Such functionality implies a membrane that is actively reconfigurable, a property which can be achieved by tuning of the geometric parameters (i.e., the length/width of the membrane segments, Figure 3a,b). An additional fixed boundary or loaded mass could be applied to the membrane to stop the vibration of a certain membrane segment. For example, consider an acoustic application. An electromagnet could electrically switch between two fixed states by firmly snapping a magnetic disc to stop membrane vibrations [41]. In the following section, the frequency tuning range of L-shaped membranes in terms of the length or width of the dimensionless segment is emphasized.

#### 3.4.1. Tunable Segment Length

**Figure 4:**
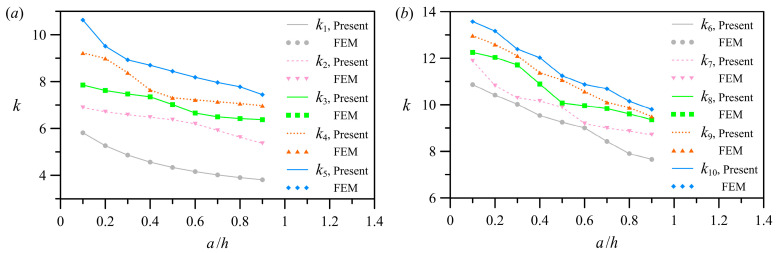
The first ten eigenvalues versus the dimensionless length *a/h* of the right-hand segment: (**a**) *k*_1_–*k*_5_; (**b**) *k*_6_–*k*_10_.

To demonstrate the impact of the dimensionless right-hand segment length (i.e., *a/h*) on eigenvalues, Figure 4 shows the computed results for the asymmetric case 2 in Table 1. Figure 4a,b corresponds to *k*_1_–*k*_5_ and *k*_6_–*k*_10_, respectively. With the increase in *a/h*, the eigenvalues gradually decrease (Figure 4a,b). The decreasing trend of the low-order eigenvalues is comparable to that of the high-order eigenvalues. Furthermore, the bandwidth of variation of the high-order eigenvalues is less than that of the low-order eigenvalues.

#### 3.4.2. Tunable Segment Width

**Figure 5:**
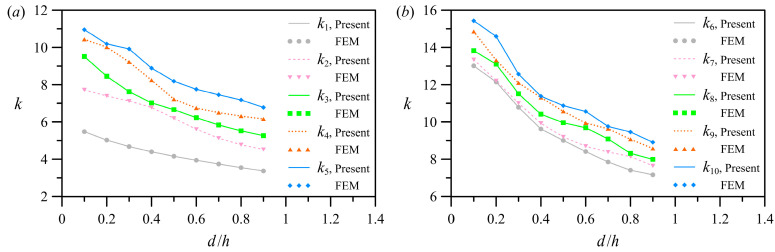
The first ten eigenvalues versus the dimensionless width *d/h* of the left-hand segment: (**a**) *k*_1_–*k*_5_; (**b**) *k*_6_–*k*_10_.

The calculated results as the left-hand segment of the L-shaped membrane becomes wider (cf. asymmetric case 3 in Table 1) are shown in Figure 5a for the first five eigenvalues and in Figure 5b for the later five ones. With the increase in *d/h*, the low-order eigenvalues decrease monotonously (Figure 5a). Figure 5b shows the sudden drop trend of the high-order eigenvalues, indicating that the high-order eigenvalues are sensitive to the width changes of the L-shaped membrane segment. In addition, similar to the observations in Figure 4, the bandwidth of variation is narrow for the high-order eigenvalues, while it is broader for the low-order eigenvalues. Notably, the width and length of the membrane segment must be actively adjusted if a high operating frequency is required.

### 3.5. Eigenmodes

The performance of membrane-based sensors is dependent on the eigenmodes of the membrane. For membrane-type actuators, specific eigenmodes are applicable to the switching between the two ground states. During the switching process, the eigenmodes are crucial for reconstructing the entire membrane trajectory [42].

**Figure 6:**
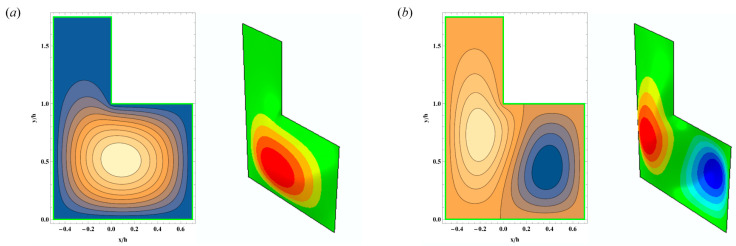

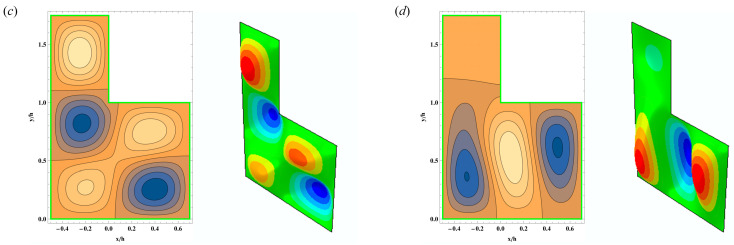
Eigenmodes for the asymmetric case 2 with *a/h* = 0.7: (**a**) 1st mode; (**b**) 2nd mode; (**c**) 5th mode; (**d**) 6th mode; left: 2D view (semi-analytical method); right: 3D view (Abaqus).

The first, second, fifth, and sixth eigenmodes are computed for the asymmetric case 2 with *a/h* = 0.7. An image sequence for these four eigenmodes is shown in Figure 6. Notably, for each of the eigenmodes, all of the transverse motions are normalized by the peak displacement value, setting the maximum displacement value to unity for each eigenmode. As expected, the first mode does not have nodal lines within the membrane (Figure 6a), while the higher modes have more nodal lines passing through the interior of the membrane (Figure 6b–d). Compared to the first mode, the higher-mode frequencies should provide more variance.

## 4. Discussion

Based on the results of previous parametric analyses, rapid identification of the membrane material in the early design stage is important for many potential applications. The key parameters of eigenfrequency (resonant frequency) and deflection (transverse displacement), which are related to membrane vibration in sensors, can affect device performance [43]. For example, the natural frequency can tune the instrument to a specific frequency range. In addition, the sensitivity of vibration-based sensors can be improved by increasing the resonant frequency [44].

### 4.1. Material Selection

**Figure 7:**
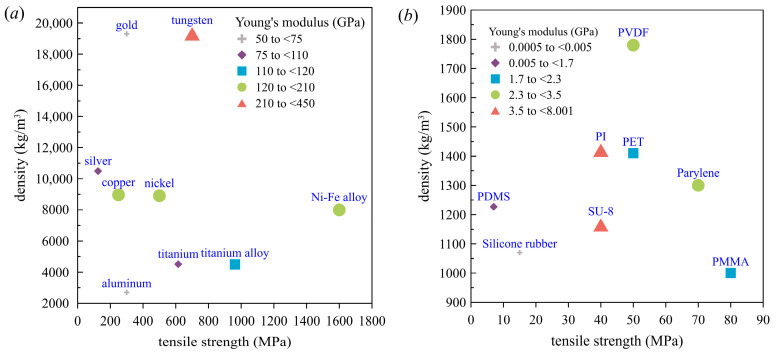
Material selection graphs for membrane-based MEMS sensors: (**a**) metals and alloys; (**b**) polymers.

When balancing or maximizing conflicting resonant frequency and membrane deflection requirements for a given vibration-based sensor, the design criteria for material selection consist of mass density, ultimate tensile strength, and Young’s modulus [45]. Figure 7a shows material selection graphs for metals and alloys for microelectromechanical systems (MEMS) materials and Figure 7b for polymers. Detailed MEMS material properties for metals and alloys are listed in Table 6 and for polymers in Table 7. To achieve a higher resonant frequency and lower membrane deflection under a given tensile force, a stiffer membrane (i.e., a higher Young’s modulus) should be selected. This can benefit measurement applications requiring high displacement resolution or increased sensitivity.

Calculations were performed for several membrane materials shown in Figure 7. The geometric conditions were the asymmetric case 1, with *h* = 1.0 mm (cf. Table 1). The applied prestress was set at 1% of the ultimate tensile strength of the selected material. The first eigenfrequencies were calculated and are listed in Table 8 for metals and alloys and Table 9 for polymers. These results may serve as a reference for operating frequencies in the design of future membrane-type sensor elements.

### 4.2. Geometry Selection

The frequency adjustment of a manufactured membrane sensor is achieved by changing the width or length of the asymmetric L-shaped membrane segments according to the mechanism shown in Figure 3. For example, consider a membrane sensor composed of a polymer material (Young’s modulus *E* = 11.9 GPa, Poisson’s ratio *v* = 0.3, and density *ρ* = 790 kg/m^3^). The membrane thickness is set to 0.1 mm, and the membrane is subjected to a biaxial prestress of 10^5^ Pa in the horizontal and vertical directions.

Table 10 summarizes the fundamental frequencies of the membrane sensors corresponding to different geometries. With a decrease in the length of the right segment, *a*, or the width of the left segment, *d*, the fundamental frequency of the membrane sensor increases. By contrast, with the increase in the length of the left segment, *b*, the fundamental frequency of the membrane sensor decreases. In addition, the vibration frequencies of the membrane sensor could be modified by changing the applied prestress of the membrane.

### 4.3. Potential Applications and Performance Sensitivity Analyses

Membrane vibration-based sensors can be designed to detect changes in resonant frequency or vibration amplitude due to external stimuli such as changes in mass, temperature, or pressure. For example, if the mass of the membrane increases or decreases due to the interaction of gas or chemicals with the membrane surface or due to other environmental factors, the natural frequency of the membrane sensor will change. As a result, the frequency shift can be used to detect the presence and concentration of gases and chemicals, as well as changes in mass [57,58,59,60].

Considering the asymmetric L-shaped membrane, we assume a 1% change in the membrane mass due to external interactions, with the added mass uniformly distributed over the entire membrane surface. The geometric parameters *b* and *h* are set to 1.75 mm and 1 mm, respectively, while the material properties of the membrane are listed in Table 2. The membrane thickness is set to 0.1 mm, and the pre-applied biaxial stress is 10^5^ Pa in the horizontal and vertical directions. We vary the geometric parameters (*a* and *d*) to test the performance sensitivity of the membrane sensor. The performance sensitivity, *S*, is defined as the ratio of the fundamental frequency shift Δ*f* to the mass change Δ*m* and is given by [58]:

(23) $$S=\frac{\Delta f}{\Delta m}$$

**Figure 8:**
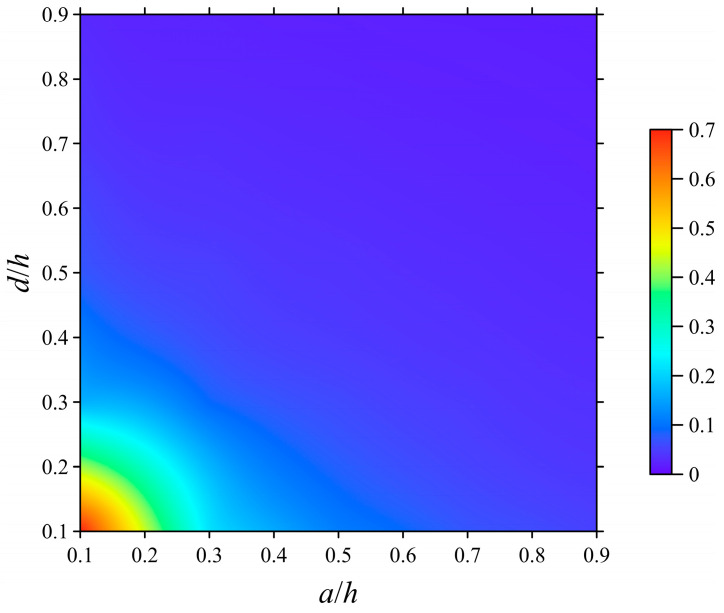
Performance sensitivities of the asymmetric L-shaped membrane sensors versus *a*/*h* and *d*/*h*.

Figure 8 demonstrates the performance sensitivities of these asymmetric L-shaped membrane sensors with varying geometric parameters. The higher the performance sensitivity, the more significant the frequency shift is under the same mass change. From Figure 8, it can be found that the performance sensitivity increases as the geometric parameters *a* and *d* are decreased. These results are due to the fact that reducing the geometric parameters increases the fundamental frequency of the membrane while decreasing the mass. Therefore, as long as there is a slight disturbance in the mass of the membrane, the shift in fundamental frequency will be easily detected.

## 5. Conclusions

In this study, a tunable vibration-based sensor comprising asymmetric L-shaped membranes was proposed for use in MEMS applications. A semi-analytical series solution was derived for the corresponding two-dimensional Helmholtz eigenvalue problem to better understand the vibration characteristics of these membranes. The region-matching technique, which combines domain decomposition and variable separation methods, was used to construct the Cartesian displacement fields. The results of this semi-analytical analysis were comparable to those of finite-element simulations over a wide range of asymmetric L-shaped geometries, as well as previous numerical (approximation) solutions for symmetric cases. These results demonstrate the good performance and computational efficiency of the semi-analytical approach.

The asymmetric L-shaped membrane sensor is advantageous as the operating frequency can be tuned by adjusting the geometry, such as the length or width of the membrane segments. The fundamental natural frequency increases with a decrease in the length or width of the membrane segments, providing a means of frequency tuning. In addition, the proposed model can be used to determine the membrane material for membrane sensors with specific frequency requirements and geometries. Based on the changes in resonant frequency due to interactions between the ambient environment and the membrane surface, these membranes could be employed as gas sensors, chemical sensors, or mass sensors. Our numerical results indicate that the performance sensitivity for mass sensing is significantly affected by the geometry of the asymmetric L-shaped membrane. The asymmetry of the membrane can thus be customized for different mass sensing sensitivity requirements.

It is worth noting that the proposed semi-analytical solutions are derived on the basis of the linear, homogeneous, and isotropic material properties of membranes subjected to a uniform tensile stress. In addition, the pre-applied tension and the material properties of the membrane are assumed to remain constant during the vibration, neglecting environmental factors, such as temperature and pressure changes. Further applications are possible if the membrane-based sensors comprise multi-segmented rectangles of different material types. In addition, fabrication issues in micromachined tunable devices will be investigated in the future.

## Figures and Tables

**Table 1:** Geometric parameters adopted for symmetric and asymmetric L-shaped membranes.

Geometric Parameters	*b*/*h*	*d*/*h*	*a*/*h*
Symmetric case	2.00	1.00	1.00
Asymmetric case 1	1.68	0.57	1.24
Asymmetric case 2	1.75	0.5	0.1–0.9
Asymmetric case 3	1.75	0.1–0.9	0.6

**Table 2:** Simulation parameters adopted for FE analysis.

Simulation Parameters	Values
Density (kg/m^3^)	750
Young’s modulus (GPa)	12
Poisson’s ratio	0.3
Uniform tensile stress (kN/m^2^)	14
Membrane thickness (mm)	0.1

**Table 3:** Nine selected eigenvalues versus truncation indices *N* for the asymmetric case 1.

*k*	*N*			
	20	200	500	2000
1	3.5408	3.5416	3.5417	3.5417
2	4.5721	4.5731	4.5732	4.5732
3	5.7980	5.7994	5.7995	5.7995
7	7.5964	7.5966	7.5966	7.5966
8	7.9849	7.9850	7.9850	7.9850
9	8.8156	8.8159	8.8159	8.8159
13	10.1352	10.1370	10.1371	10.1371
14	10.5901	10.5916	10.5917	10.5917
15	10.7652	10.7652	10.7652	10.7652

**Table 4:** First ten eigenvalues for the symmetric case.

*k*	Present	Abaqus	Fantuzzi et al. [37]	Katsikadelis and Sapountzakis [17]	Fox et al. [25] *
1	3.1048	3.1049	3.1046	3.101	3.1048
2	3.8984	3.8983	3.8984	3.896	3.8984
3	4.4429	4.4430	4.4429	4.438	4.4429
4	5.4334	5.4333	5.4334	5.423	5.4334
5	5.6491	5.6491	5.6489	5.617	5.6491
6	6.4401	6.4401	6.4399	6.399	6.4401
7	6.7044	6.7044	6.7044	6.677	6.7043
8	7.0248	7.0249	7.0248	7.020	7.0248
9	7.0248	7.0249	7.0248	7.051	7.0248
10	7.5306	7.5307	7.5305	7.486	7.5306

Note: The asterisk indicates all of the values in the literature are square-rooted.

**Table 5:** First fifteen eigenvalues for the asymmetric case 1.

*k*	Present	Abaqus
1	3.5417	3.5417
2	4.5732	4.5733
3	5.7995	5.7995
4	6.2459	6.2460
5	6.6358	6.6357
6	7.1692	7.1693
7	7.5966	7.5967
8	7.9850	7.9850
9	8.8159	8.8160
10	9.2079	9.2080
11	9.3786	9.3786
12	9.6741	9.6741
13	10.1371	10.1372
14	10.5917	10.5918
15	10.7652	10.7653

**Table 6:** MEMS material properties for metals and alloys.

Materials	Density (kg/m^3^)	Young’s Modulus (GPa)	Tensile Strength (MPa)	Ref.
nickel	8900	207	500	[45]
aluminum	2700	70	300	[46]
copper	8960	120	250	[47]
gold	19,300	70	300	[46]
titanium	4510	105	615	[48]
tungsten	19,300	410	700	[48]
Ni-Fe alloy	8000	120	1600	[46]
titanium alloy	4500	110	962	[49,50]
silver	10,490	83	125	[46,51]

**Table 7:** MEMS material properties for polymers.

Materials	Density (kg/m^3^)	Young’s Modulus (GPa)	Tensile Strength (MPa)	Ref.
PI	1420	8	40	[48]
SU-8	1164	3.5	40	[48]
Parylene	1300	3	70	[49]
Silicone rubber	1070	0.0005	15	[52,53]
PVDF	1780	2.3	50	[47]
PMMA	1000	2	80	[46]
PDMS	1227	0.005	7	[54,55]
PET	1410	1.7	50	[56]

**Table 8:** First eigenfrequencies for sensing membranes comprising metals or alloys selected from Figure 7a.

Materials	Frequency *f*_1_ (Hz)
Nickel	13,361
Titanium	20,815
Ni–-Fe alloy	25,209
Titanium alloy	26,062

**Table 9:** First eigenfrequencies for sensing membranes comprising polymers selected from Figure 7b.

Materials	Frequency *f*_1_ (Hz)
Polydimethylsiloxane (PDMS)	4258
Polyvinylidene difluoride (PVDF)	9447
Polyimide (PI)	9461
Polyethylene terephthalate (PET)	10,615

**Table 10:** First eigenfrequencies for sensing membranes with different geometries.

Case	*a* (mm)	*b* (mm)	*d* (mm)	*h* (mm)	Frequency *f*_1_ (Hz)
1	1.24	1.68	0.57	1.0	6342
2	1.00	1.68	0.57	1.0	6550
3	1.24	1.68	0.35	1.0	6630
4	1.00	1.68	0.35	1.0	6977
5	1.24	1.50	0.57	1.0	6343

## Data Availability

The data presented in this study are available on request from the corresponding author.
